# ADNP is essential for sex-dependent hippocampal neurogenesis, through male unfolded protein response and female mitochondrial gene regulation

**DOI:** 10.1038/s41380-024-02879-w

**Published:** 2024-12-23

**Authors:** Guy Shapira, Gidon Karmon, Gal Hacohen-Kleiman, Maram Ganaiem, Shula Shazman, Paschalis Theotokis, Nikolaos Grigoriadis, Noam Shomron, Illana Gozes

**Affiliations:** 1 Department of Cell and Developmental Biology, Faculty of Medical and Health Sciences, Sagol School of Neuroscience, Edmond J Safra Center for Bioinformatics, Tel Aviv University, Tel Aviv, 6997801 Israel; 2 Elton Laboratory for Molecular Neuroendocrinology, Department of Human Molecular Genetics and Biochemistry, Faculty of Medical and Health Sciences, Adams Super Center for Brain Studies and Sagol School of Neuroscience, Tel Aviv University, Tel Aviv, 6997801 Israel; 3 Department of Mathematics and Computer Science, The Open University of Israel, Ra’anana, 4353701 Israel; 4 Department of Neurology, Laboratory of Experimental Neurology, AHEPA University Hospital, Aristotle University of Thessaloniki, Thessaloniki, Greece

**Keywords:** Molecular biology, Neuroscience, Autism spectrum disorders

## Abstract

Essential for brain formation and protective against tauopathy, activity-dependent neuroprotective protein (ADNP) is critical for neurogenesis and cognitive functions, while regulating steroid hormone biogenesis. As such, de novo mutations in ADNP lead to syndromic autism and somatic ADNP mutations parallel Alzheimer’s disease progression. Furthermore, clinical trials with the ADNP fragment NAP (the investigational drug davunetide) showed efficacy in women suffering from the tauopathy progressive supranuclear palsy and differentially boosted memory in men (spatial) and women (verbal), exhibiting prodromal Alzheimer’s disease. While autism is more prevalent in boys and Alzheimer’s disease in women, both involve impaired neurogenesis. Here, we asked whether ADNP sex-dependently regulates neurogenesis. Using bromodeoxyuridine (BrdU) as a marker of neurogenesis, we identified two-fold higher labeling in the hippocampal sub-ventricular zone of ADNP-intact male versus female mice. *Adnp* haplo-insufficient (*Adnp*^*+/−*^) mice or mice CRSIPR/Cas9-edited to present the most prevalent neurodevelopmental ADNP syndrome mutation, p.Tyr718* (Tyr) showed dramatic reductions in male BrdU incorporation, resulting in mutated females presenting higher labeling than males. Treatment with NAP compensated for the male reduction of BrdU labeling. Mechanistically, hippocampal RNAseq revealed male-specific Tyr down-regulation of endoplasmic reticulum unfolded protein response genes critical for sex-dependent organogenesis. Newly discovered mitochondrial accessibility of ADNP was inhibited by the Tyr718* mutation further revealing female-specific Tyr downregulation of mitochondrial *ATP6*. NAP moderated much of the differential expression caused by p.Tyr718*, accompanied by the down-regulation of neurotoxic, pro-inflammatory and pro-apoptotic genes. Thus, ADNP is a key regulator of sex-dependent neurogenesis that acts by controlling canonical pathways, with NAP compensating for fundamental ADNP deficiencies, striding toward clinical development targeting the ADNP syndrome and related neurodevelopmental/neurodegenerative diseases.

## Introduction

Our past studies indicated that activity-dependent neuroprotective protein (ADNP)-deficient embryos exhibit dramatic increases in mRNA species associated with lipid metabolism, coupled with a reduction in the levels of organogenesis/neurogenesis-related transcripts [1]. We first cloned, identified and characterized ADNP from the pluripotent teratocarcinoma P19 cell line induced to differentiate into neuroglial-like cells [2]. We further showed that ADNP interacts with specific chromatin regions in the neuro-differentiated state, and that ADNP is directly bound to heterochromatin protein 1 (HP1alpha). These studies were extended to describe interactions of ADNP with multiple other chromatin-regulating proteins, which were coupled with direct DNA-protein interactions [1–4]. Focusing on neurogenesis, we showed that ADNP regulates neurogenin1 (neurog1), neuroD1 (neuroD1), and beta3 tubulin [1, 5]. We then identified ADNP-mediated regulation of the WNT signaling pathway [6]. These studies were subsequently verified and extended to show that ADNP stabilizes a key player in this signaling pathway, namely, β-catenin, through binding to the β-catenin armadillo domain, which then prevents association with critical components of the degradation complex, specifically, axin and adenomatous polyposis coli (APC). Interestingly, davunetide, an investigational drug corresponding to the NAP (NAPVSIPQ) motif of ADNP mediates the association with β-catenin [7, 8].

In deciphering the transcriptional activities of ADNP, we reported that ADNP regulates sex steroid biogenesis [9]. Accordingly, several studies have described sex-dependent hippocampal neurogenesis, showing direct effects of steroid hormones on adult neurogenesis [10], as well as on stress [11] and spatial learning [12]. Indeed, mutations and aberrations in *ADNP* expression are associated with stress, autism, intellectual disability (ADNP syndrome, also known as Helsmoortel Van Der Aa syndrome), cognitive dysfunction, Alzheimer’s disease [6, 9, 13, 14], and schizophrenia [15], as well as Parkinson’s disease [16] and muscle disorders [17], showing sexual dichotomy in humans [14, 15, 17, 18] and mouse models [17, 19–23] and emphasizing the importance of identifying mechanism of action and interactions of ADNP.

Our earlier efforts connected ADNP to embryogenesis through the regulation of markers of neurogenesis (i.e., Ngfr, neurogenin1, and neurod1) and of heart development (Myl2) [1]. We further identified the growth regulator AKT, that in turn controls WNT signaling, as a key protein controlled by *Adnp* haploinsufficiency [21] that is accentuated in female spleens in mice heterozygous for the mouse p.Tyr718* homologue of the most abundant ADNP syndrome mutation, p.Tyr719* (Tyr mice [22]).

Remarkably, previous results suggested differential epigenetic signatures in the *A*DNP p.Tyr719* and neighboring mutations in humans (corresponding to our Tyr mouse model) and other *Adnp* mutations (potentially corresponding to haploinsufficiency) [24]. These findings were further corroborated by our genome-edited cell culture system that revealed a more severe gain-of-toxic-function for the Tyr mutation, as opposed to a mutation potentially mimicking haploinsufficiency [25]. Similarly, our Tyr mouse exhibited gain-of-toxic-function with early onset of Tau deposits and more severe motor impairments [22].

Here, using our two complementary mouse models, we compared the direct effect of *Andp* on neurogenesis in conjunction with hippocampal gene expression. We used bromodeoxyuridine (BrdU), a thymidine analog that is incorporated into the DNA of dividing cells during S-phase of the cell cycle, allowing for neuronal birth dating, and monitoring of cell proliferation so as to study neurogenesis in either *Adnp* haploinsufficient or Tyr mice. We discovered *Adnp*-dependent differential regulation of neurogenesis in males and females and revealed unfolded protein response dysregulation in the hippocampal endoplasmic reticulum (ER) specific to Tyr male mice. We further discovered *Adnp* mitochondrial bioavailability, coupled with regulation of Tyr mouse female mitochondrial gene expression, hampered by *Adnp* mutation, and compensated by NAP treatment.

## Methods

### Adult mouse neurogenesis: BrdU incorporation

Two and half month-old *Adnp*^*+/−*^ mice on ICR background and Tyr mice (heterozygous for *Adnp* p.Tyr718*) on C57BL6/NJ background, were handled as described [21, 22]. The mice were habituated at the same time under the exact same conditions. At 4 weeks of age, the mice were daily treated intranasally with 0.5 µg NAP or vehicle (DD, in which each milliliter included 7.5 mg of NaCl, 1.7 mg of citric acid monohydrate, 3 mg of disodium phosphate dihydrate and 0.2 mg of a 50% benzalkonium chloride solution) for 5−6 weeks [21, 22]. The mice were then injected (i.p.) with BrdU (80 mg/kg) at 2 h intervals, as previously described [26], and euthanized an hour after the last injection. Tissues were processed as previously described [21, 22].

### BrdU immunohistochemistry

Immunofluorescence for BrdU detection was performed as previously described [27] and further detailed in the supplementary methods.

### Hippocampal RNA-seq

RNA was extracted from the hippocampus of 2.5-month-old Tyr mice, treated, handled, and sequenced on a NextSeq500 apparatus (Illumina, San Diego, CA), and analyzed as previously described [22]. Six groups of mice (wild type, Tyr- and Tyr-treated with NAP, separated by sex) were used, with each treatment including at least three biological replicates. Data sets from our previous works [19, 21], as well as other published data sets [28] were also analyzed.

### Mitochondria labeling

Neuronal-like differentiated mouse neuroblastoma N1E-115 cell clones, CRISPR/Cas9-edited to express green fluorescent protein (GFP)-labeled full-length ADNP and GFP-labeled ADNP p.Tyr718* [25, 29], were incubated with MitoTracker deep red FM (M22426, Invetrogen Thermo Fisher Scientific, Waltham, MA) at a concentration of 250 nM for 30 min in 95% air/5% CO_2_ in a humidified incubator (37 ^0^C) (https://tools.thermofisher.com/content/sfs/manuals/mp07510.pdf). Co-localization of two fluorophores was quantified as before [16].

### Bioinformatics analysis

Raw sequencing data was trimmed and filtered using fastp [30], followed by transcript quantification with Salmon [31] according to the GRCm38 reference genome, with length and positional corrections enabled. All additional bioinformatics tools are delineated in the supplementary methods.

## Results

### Higher male/female neurogenesis is regulated by the major neurodevelopmental/intellectual disability/autism-linked ADNP

**Fig. 1:**
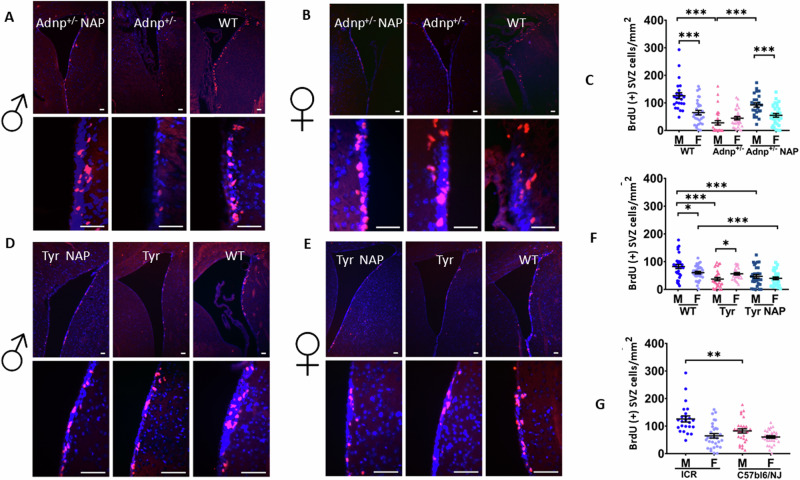
Comparison of BrdU-labeled cell concentrations in the SVZ of two ADNP mouse models, *Adnp*^+/−^ on ICR background and Tyr mice on C57BL6/NJ background. **A**–**C** *Adnp*^+/−^ mice, (**D**–**F**) Tyr mice, (**G**) wild type, ICR and C57BL6/NJ comparisons. (**A**, **B**, **D**, **E**) Representative images (scale bar = 100 µm). **C**, **F**, **G** Group differences in BrdU-positive cells/mm2 (mean ± SEM) were compared using a one-way analysis of variance with Tukey’s post-hoc test. Technical replicates (six per animal) were used for statistical analysis. Outliers were excluded based on Grubb’s test. **C** For *Adnp*^+/−^ males, a statistically significant difference for BrdU-positive cells was discovered (F_2,77_ = 33.030, *p* < 0.001), with Tukey’s post-hoc test revealing a significant reduction in BrdU-positive cells in *Adnp*^+/−^ DD-treated males (*N* = 4), as compared to WT (*N* = 5) (***P < 0.001), which was significantly corrected upon NAP treatment (***P < 0.001, *N* = 5). No such effect was found in females (*N* = 5 per group). Significant sex differences were discovered in the WT and the NAP-treated *Adnp*^+/−^ group (***P < 0.001 for both comparisons). **F** For Tyr females, a statistically significant difference for BrdU-positive cells was discovered (F_2,77_ = 11.272, ***P < 0.001), with Tukey’s post-hoc test revealing a significant reduction in BrdU-positive cells in NAP-treated Tyr females (*N* = 5), as compared to WT (*N* = 4) (***P < 0.001), with no difference being seen when compared to DD-treated Tyr females (*N* = 5). For Tyr males, a statistically significant difference for BrdU-positive cells was discovered (F_2,89_ = 12.777, ***P < 0.001), with Tukey’s post-hoc test revealing a significant reduction in BrdU-positive cells in DD- and NAP-treated Tyr males (*N* = 5 for both groups), as compared to WT (*N* = 5) (***P < 0.001). Significant sex differences were discovered in the WT and the DD-treated Tyr groups (*P* < 0.05 for both comparisons). **G** Significant differences were discovered among males of the different tested strains (*P* < 0.01).

Two-fold higher BrdU labeling was noted in the hippocampal sub-ventricular zone (SVZ) of *Adnp*^*+/+*^ male mice, as compared to *Adnp*^*+/+*^ female mice (wild type, WT, ICR background), as depicted in Fig. 1A, B (BrdU immunohistochemistry) and Fig. 1C, showing densitometry results of 4-5 different animals, each depicting left and right hippocampi/condition. Similarly, two-fold higher BrdU labeling was also apparent in WT C57BL6/NJ male mice compared to female mice (Fig. 1D−F). Interestingly, the mouse strain used affected BrdU labeling, as exemplified with ICR males that showed 1.5-fold higher incorporation, as compared to C57BL6/NJ mice (**P < 0.01, Fig. 1G). *Adnp* haploinsufficiency (*Adnp*^*+/−*^, ICR background) or CRSIPR/Cas9 editing to generate the most prevalent neurodevelopmental ADNP syndrome mutant, i.e., the mouse equivalent p.Tyr718* (heterozygous Tyr mice with a C57BL6/NJ background) showed dramatic reduction in BrdU incorporation, resulting in mutated females that presented higher BrdU labeling than males (*P < 0.05, Tyr mice, Fig. 1F). NAP treatment resulted in significantly increased BrdU incorporation by *Adnp*^*+/−*^ male mice, while in Tyr mice, only a trend was observed (possibly due to background strain effects, as illustrated above), coupled with a significant decrease in NAP-treated Tyr females, relative to WT C57BL6/NJ animals (Fig. 1).

### Distinct hippocampal gene expression and differential transcript levels induced by the ADNP Tyr mutation and NAP treatment

**Fig. 2:**
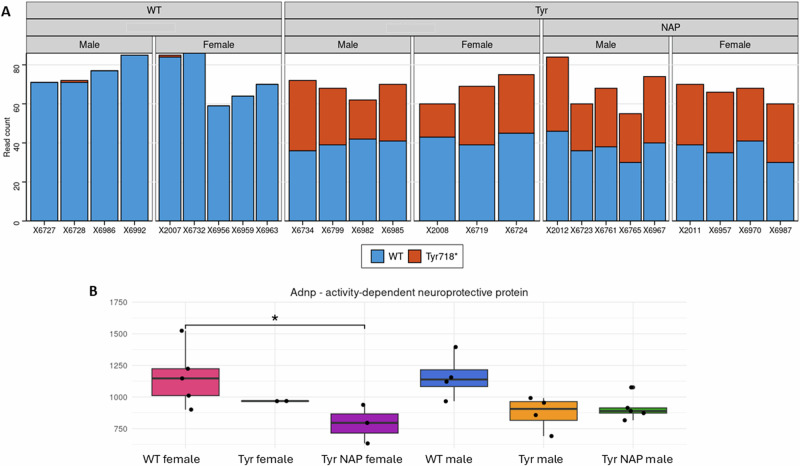
Heterozygous *Adnp* expression in Tyr mice. **A** Bar plot of hippocampal *Adnp* expression in WT and Tyr mice, treated by vehicle or NAP as before [22] by allele (read count) and (**B**) boxplot of total *Adnp* gene expression (normalized expression). Note that allelic expression is based solely on reads overlapping the Tyr718* mutation locus, while overall gene expression is based on all reads overlapping the gene, regardless of genotype.

Analysis of RNA sequencing data from hippocampal samples revealed that about 50% of the *Adnp* transcripts from Tyr mice were mutated, regardless of sex or treatment (Fig. 2A), in agreement with the heterozygous genotype and results from our previous spleen-based study [22]. Total *Adnp* gene expression was slightly down-regulated in the mutated groups, paralleling BrdU incorporation levels (Fig. 1F), although the difference was statistically significant only in NAP-treated Tyr females (Fig. 2B).

**Fig. 3:**
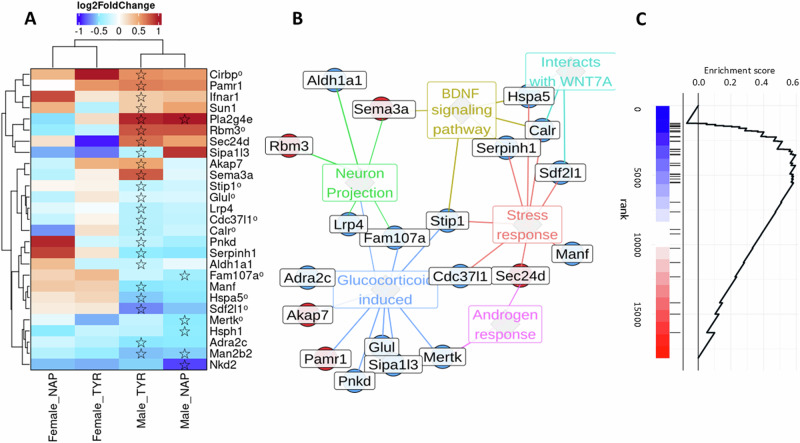
*Adnp*-specific regulated expression in Tyr males. **A** Heatmap of differential expression colored according to log2-fold change for each gene (row) at each comparison (column). In cases of significant differential expression (FDR < 0.05), the cell is marked with a star. If differential expression was only significant at the transcript level, the gene name is marked with a circled suffix. **B** A graph of relationships between differentially expressed genes (small nodes) and terms with which they are associated. Gene nodes are marked red (up-regulation) or blue (down-regulation) according to their significant differential expression trend. **C** GSEA plot of genes up-regulated by the HSP-90 inhibitor geldanamycin according to their differential expression in the Tyr male comparison. The position of the enriched genes is marked in a ranked list at the bottom, starting from the most downregulated (blue) to the most up-regulated (red).

Overall *Adnp* mutation-related differential expression was almost entirely distinct between males and females, with only few genes similarly differentially expressed in both sexes and/or across treatments (Table S1). In addition to differentially expressed genes (DEGs), we found many differentially expressed transcripts (DETs), suggesting an effect on RNA splicing, most prominently in the Tyr groups (Table S2). The differential expression of mRNA splicing regulators, such as the X-chromosome-linked cold-response protein RNA-binding motif 3 (*Rbm3*) and the cold-inducible RNA-binding protein (*Cirbp*; Fig. 3) in males, might underlie some of the extensive differential splicing seen.

### Unfolded protein response (UPR) genes are downregulated in male Tyr hippocampus, yet maintained expression upon NAP treatment

In males, the Tyr mutation induced 34 DEGs and 23 DETs, while NAP treatment largely resulted in maintained expression, with just 10 DEGs and 6 DETs appearing. A large set of genes associated with ER-driven cellular stress response, commonly referred to as UPR genes, were exclusively down-regulated in Tyr males, an effect that was either weakened or rendered insignificant upon NAP treatment (Fig. 3A, B). Gene set enrichment analysis indicated these to be part of a broader down-regulated HSP-90-inhibited transcriptional signature of 26 genes. Genes induced by the HSP90 inhibitor geldanamycin were strongly down-regulated in male TYR mice (NES = −2.59; FDR<1e-5) (Fig. 3C), including the HSP90 co-chaperones *Cdc37l1* and *Stip1*.

Hippocampal expression of UPR genes is essential for long-term memory formation, with their disruption having been recently shown to impair learning [32]. In the same study, down-regulation of UPR genes was induced by dysregulation of *Nr4a* and reversed by over-expressing *Hspa5*, which was also down-regulated at the transcript level in Tyr males (Fig. 3A). While we found no significant expression difference in genes of the *Nr4a* family, there was a significant enrichment of down-regulated glucocorticoid-induced genes (FDR<1e-4, Fig. 3B), with both glucocorticoid and stress being known to regulate NR4A [33]. We also found down-regulation of neuroprotective genes, such as *Manf* and *Sipa1l3*, which was also prevented by NAP treatment, resulting in neurological benefit in the hippocampi of old mouse models [34]. The genes *Sun1* and *Ifnar1*, up regulated in Tyr male mice and unchanged in level by NAP treatment, are associated with neuronal injury and potential disruption of synaptic plasticity [35, 36].

NAP-induced down-regulation of various ER stress genes in males, including those encoding the pro-neuroinflammatory *Nkd2* [37], *Hsph1* (Hsp110/105) and *Fam107a* (DRR1), which were shown to exacerbate cerebral ischemia [38], and *Mertk*, the mediator of alpha-synuclein fibril uptake [39]. A few male Tyr DEGs were unchanged in level regardless of NAP treatment, including Tyr up-regulation of *Pla2g4e*, which is associated with cognitive resilience in late-onset Alzheimer’s disease models [40] and down-regulation of the glycosylation-related *Man2b2* [41].

### Differential expression in Tyr-mutated females and the divergent NAP transcriptional signature

**Fig. 4:**
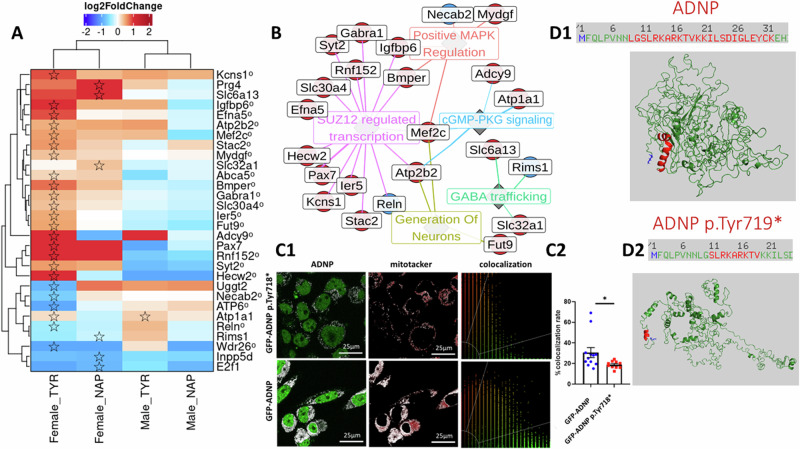
*Adnp*-specific regulated expression in Tyr females. **A** Heatmap of differential expression colored according to log2-fold change for each gene (row) at each comparison (column). In cases of significant differential expression (FDR < 0.05), the cell is marked with a star. If differential expression was only significant at the transcript level, the gene name is marked with a circled suffix. **B** A graph of relationships between differentially expressed genes (small nodes) and terms with which they are associated. Gene nodes are marked red (up-regulation) or blue (down-regulation) according to their significant differential expression trend. **C1** Mouse neuroblastoma N1E-115 cell clones expressing CRISPR/Cas9-edited full-length ADNP or heterozygous ADNP p.Tyr718* [25, 29] were endogenously stained with MitoTracker (red)-. Co-localization of ADNP and MitoTracker is represented by white dots. Quantitative analysis of ADNP/MitoTracker merged staining, reflecting co-localization, is presented in the graph; images were viewed using a x63 oil immersion lens. **C2** Statistical analysis of the co-localization rate calculated by addressing merged staining in a Leica sp8 fluorescent microscope. A two-tailed t-test confidence level of 95% was determined using PRISM Statistics software, version 24 (IBM, Armonk, NY), *P < 0.05. **D** ADNP contains a mitochondrial targeting sequence [58, 59] ADNP is represented in dark green, while the internal mitochondrial targeting sequence is colored red (**D1**). The sequence is shortened in ADNP p.Tyr719*, with further structural differences as previously highlighted [8] (**D2**). ADNP and ADNP p.Tyr719* structures were retrieved using the I-TASSER server. The figures were created using PyMOL software as before [8, 14].

In females, the Tyr mutation induced 43 DETs and 4 additional DEGs, while NAP treatment generated 27 DEGs and 9 DETs (Fig. 4A). The genes affected by DET exclusive to Tyr females were enriched in those associated with various neurodegenerative diseases. These included numerous individual genes associated with neurodevelopmental disorders, such as *Wdr26*-associated intellectual disability [42], *Col6a2*-associated behavioral and cortical dysfunction [43], and a wide range of Hecw2-linked neurodevelopmental disorders [44], as well as the crucial corticogenesis gene *Reelin* [45].

Enrichment analysis found that differentially expressed genes and gene transcripts in female Tyr mice were disproportionately associated with schizophrenia [46], dysregulation of glial cell transcriptome in autism [47] and GABAergic genes conferring neuropsychiatric disorder risk [48] (gene set enrichment results; Table S3). Differentially expressed ATP metabolism genes, such as that of the mitochondrially-encoded ATP synthase membrane subunit 6 (*mt-ATP6*), *Atp2b2* and *Abca5*, are associated with neuropathologies/neurodegenerations, like Alzheimer’s disease and autism [49–52].

NAP treatment either prevented or moderated nearly all Tyr-induced DE in females to statistical insignificance, as compared to controls, while inducing a distinct transcriptional signature. NAP treatment up-regulated GABA vesicle transporters *Slc32a1*, *Slc6a13*, and the anti-neuroinflammatory *Prg4* [53], while down-regulating genes for pro-apoptotic *E2f1* [54] and the microglial inflammasome activator *Inpp5d* [55] (Fig. 4A, B).

While genes from the previously mentioned UPR pathway were not differentially expressed in females, *Uggt2*, which functions as a misfolded protein sensor for UPR activation, was downregulated in female Tyr mice [56]. Tyr female genes were especially enriched in targets of the SUZ12 transcription factor (Fig. 4B) (FDR < 0.05; Table S3), which is a vital regulator of neurogenesis both in the embryonic and adult stages [57].

We further addressed whether the ADNP enters mitochondria to potentially regulate mitochondrial gene expression. To answer this question, we utilized genome-edited GFP-ADNP and GFP-ADNP p.Tyr718* cells [25, 29] stained with MitoTracker. We found extensive mitochondrial ADNP localization, which was reduced in the Tyr-mutated cells (Fig. 4C1, C2). These observations agree with a significant change in the alternative splicing of *mt-ATP6* (Fig. 4A).

Interestingly, modeling the human ADNP (90% identical to the mouse sequence at the RNA level [4]) and ADNP p.Tyr719* structures (homologous the mouse sequence above) identified an N-terminal α-helical basic amino acid-enriched mitochondrial targeting sequence [58, 59] in human ADNP (Fig. 4D1), with identical sequence in mouse [4]. This α-helical structure was found to be shortened in the human ADNP p.Tyr719* mutant molecule (Fig. 4D2), explaining the biological results (Fig. 4C1, C2) and translatable to the human condition.

### Comparative analysis of ADNP mutant models and protocadherin gene expression

**Fig. 5:**
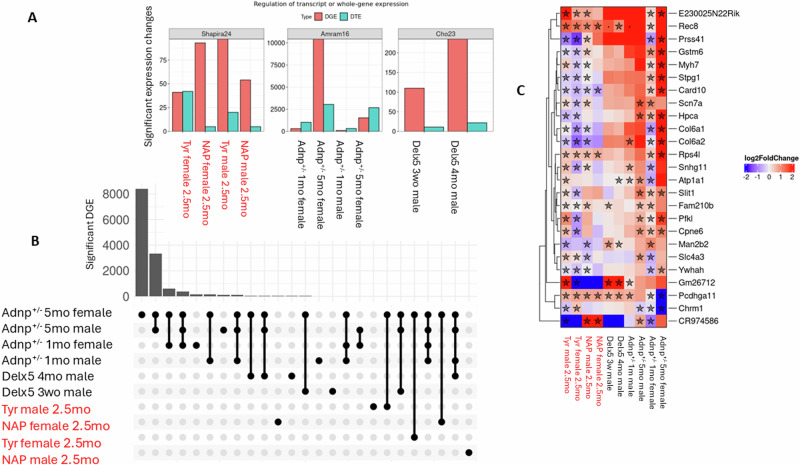
Differential expression across multiple RNA-seq experiments revealing variations according to *Adnp* mutation and mouse model. **A** The number of genes with significant differential expression across comparisons, either at the whole gene (DGE) or the single transcript level (DTE). **B** Upset plot of significant differentially expressed genes (FDR < 0.05), where each column is representative of a subset of genes from one or more comparisons (represented by rows). The size of each gene subset is visualized in the bar-plot in the top panel, with both subsets (columns) and comparisons (rows) sorted from the most to the fewest genes. **C** Heatmap of genes significantly differentially expressed (FDR < 0.05) in five or more comparisons. Significant results (FDR < 0.05) are marked with a star, while near-significant results (0.05 < FDR < 0.1) are marked with a dot.

We further compared differential hippocampal expression of our heterozygous Tyr model (prefix Tyr or NAP, depending on the treatment in Fig. 5 and Table S3), heterozygous deletion of exon 5 of *Adnp* from Cho et al. [28] (prefix Delx5) and our heterozygous deletion of *Adnp* evaluated by Amram et al. [19] (prefix Adnp^+/−^). The magnitude of differential expression was highly sex- and age-specific and roughly correlated with the severity of the mutation, with the haploinsufficient variant being the most severe, and Tyr being the mildest. Some groups had a high proportion of DTE, suggesting an effect partially mediated by differential splicing (Fig. 5A). The most differentially expressed genes were shared in comparisons of the same mutation, followed by comparisons of the same age and sex (Fig. 5B).

The relatively few differentially expressed genes that were common to five or more comparisons (including at least one from our Tyr models) were enriched with axon guidance genes (i.e., those encoding *Col6a2*, *Col6a1*, *Slit1* and *Scn7a*), thyroid hormone signaling genes (i.e., those encoding *Pfkl*, *Atp1a1* and *Myh7*), and PI3K-AKT signaling genes (i.e., *Chrm1*, *Col6a2*, *Col6a1* and *Ywhah*) (Fig. 5C). The most common differentially expressed gene by far was that of protocadherin gamma A11 (*Pcdhga11*), which was uniformly up-regulated by 50% (FDR<1e-10) in all comparisons of Delx5 and Tyr models, regardless of treatment (Fig. 5). However, in the adult *Adnp*^+/−^ females, a contrasting decrease was observed. Many *pcdh* genes were further differentially expressed due to the Tyr mutation (Table S1). For example, *Pcdhga9* was decreased by 50%, but not in NAP female (Table S1).

## Discussion

**Fig. 6:**
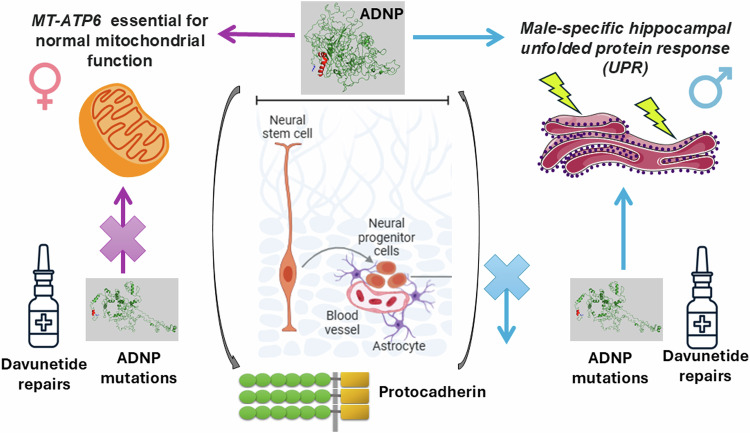
ADNP is essential for sex-dependent hippocampal neurogenesis, through male unfolded protein response and female mitochondrial gene regulation, schematic representation. The scheme shows the discovery of ADNP’s association with increased neurogenesis in males that is reduced in the face of ADNP deficiencies and corrected in part by NAP (davunetide) treatment. It further depicts the discovery of the differential ADNP/NAP regulation of the unfolded protein response in males versus the regulation of the essential MT-ATP6 in females, corrected by NAP (davunetide) treatment. Lastly, the newly revealed involvement of protocadherin in ADNP function is highlighted.

Our results suggest that ADNP content serves as a key regulator of increased male neurogenesis, through UPR genes. In other words, male neurogenesis deficits induced by the Tyr mutation were mediated by mostly separate sex-specific pathways and by partial alleviation by NAP, as underlined by prevention of ADNP Tyr mutation-induced differential expression, as well as through possible compensation by induced expression of neuroprotective factors. We also discovered female-specific mitochondrial RNA alternative splicing regulation by ADNP, as exemplified by *mt-ATP6*. We further propose the involvement of protocadherin genes that are commonly differentially expressed in the hippocampus of multiple *Adnp* loss-of-function models, regardless of age, sex, or treatment, suggesting a consistent functional link that is seemingly unaffected by NAP treatment (Fig. 6, schematic summary).

Previous results suggesting mutation-dependent ADNP epigenetic sginatures [24], were translated here into sex/mutation differential effects of ADNP/NAP on gene expression and neurogenesis. Interestingly, we implied increased hippocampal *ADNP* expression in males versus females [20], which may be mouse strain dependent. We further measured an *Adnp*^+/−^ female-specific decrease in splenic *Adnp* expression, which was ameliorated by NAP treatment in 19–27-day-old mice [21]. A similar change was not, however, observed in the hippocampus, accentuating age-, sex-, tissue- and mutation–specific ADNP expression patterns, including those subject to estrus-cycle regulation [60], as well as auto-regulation [1].

Here, we indicated that the Tyr mutation caused a significant downregulation of numerous hippocampal UPR genes exclusively in males, which was either moderated or prevented by NAP treatment. This down-regulation of male UPR signaling might contribute to the substantial decrease in hippocampal neurogenesis, since this signaling system is a pivotal regulator of neurodevelopment [61] in a sex-dependent manner [62]. Indeed, the sexually dimorphic nature of the UPR is well known and apparent even at the placental stage [63]. Some down-regulated UPR genes had higher hippocampal expression in males at around 2 months of age [64] (a difference that was insignificant when compared with our WT animals), correlating with neurogenesis rates being higher in WT, yet lower in mutated males, as compared with their female counterparts. Past studies suggested that these sexual differences stem from UPR modulation by estrogen signaling [65], which is also known to regulate neuronal actin polymerization [66].

The UPR is also involved in a broad spectrum of disorders associated with ADNP dysfunction [67], including autism [68], schizophrenia [69], Alzheimer’s disease [70] and other late-onset neurodegenerative disorders [71]. It is important to note that UPR function is highly contextual. Thus, while hippocampal up-regulation of the UPR is essential for some cognitive functions [32], it is also highly deleterious in the contexts of obesity and brain trauma [72, 73].

The limited, albeit distinct differential expression induced by NAP treatment in males might represent a form of indirect compensation, in addition to the prevention or moderation of Tyr-induced differential expression. Interestingly, the UPR is activated in disease-affected brain regions in Alzheimer’s disease and progressive supranuclear palsy (PSP) [74]. Indeed, we have shown female-specific davunetide (NAP)-mediated protection in a PSP clinical trial [18]. Moreover, in patients suffering from amnestic mild cognitive impairment, prodromal to Alzheimer’s disease, davunetide boosted spatial memory in men and verbal memory in women [75]

Here, we further discovered differential expression of mRNA splicing regulators, such as X-*Rbm3* and *Cirbp* (Fig. 3, males), involving extensive differential splicing, with female Tyr vs. WT showing 43 DETs and 4 additional DEGs, while NAP treatment led to the appearance of 27 DEGs and 9 DETs. In Tyr males, the picture was reversed, with 34 DEGs and 23 DETs appearing, and NAP treatment largely maintaining expression levels, with just 10 DEGs and 6 DETs being seen. In this respect, immunoprecipitation-based studies suggested a BRM-ADNP interaction coupled to ADNP-polypyrimidine tract-binding protein (PTB)-associated splicing factor (PSF) binding, with PSF being a direct regulator of *Tau* transcript splicing [76] and with Tau deposition (tauopathy) being noted in both *Adnp*^*+/−*^ [77] and Tyr mice [22], as well as in ADNP syndrome post-mortem tissue [9]. Interestingly, no alternative splicing was detected in our spleen RNAseq study of Tyr mice [22], whereas dysregulated brain alternative splicing has been tightly associated with autism [78]. Furthermore, aging was found to increase chromatin accessibility in the male and female hippocampus, especially in repetitive elements, reflected as an increase in LINE-1 transcription [79], which has been linked to ADNP in determining heterochromatin nanodomains of methylation [80]. Significant sex-bias in chromatin accessibility in both autosomes and on the X-chromosome has been reported, with aging male-biased accessibility enriched at promoters and CpG-rich regions [79]. This is further confounded with ADNP p.Tyr719* association with hyper-methylation of CpG-rich regions, explaining the apparent accelerated aging linked with *ADNP* mutants [14]. Additionally, we have implicated ADNP-WDR5 binding [16], and in turn, WDR5 mediated regulation of stemness is associated with estrogen control [81].

Our discovery of ADNP female-specifically regulating *mt-ATP6* splicing/expression reveals ADNP mitochondrial activity that is associated with reduced Adnp pTyr718* mitochondrial bioavailability, with mt-ATP6 playing a crucial role in oxidative stress regulation [49]. Importantly, global variability in gene expression and alternative splicing is modulated by mitochondrial content [82]. With cytoskeletal health regulated by NAP, playing a major role in mitochondrial function [83], and with NAP-mediated correction of *mt-ATP6* splicing/expression, our results pave the path to NAP (davunetide)-linked sex-specific development. Importantly, the multifaceted role of mitochondria intimately entwined with neuroprotection/neurodegeneration was recently reviewed strongly implicating breakdown of function as a major contributor to abnormal brain development leading to ASD [84] including the ADNP syndrome [85]. Interestingly, Rett syndrome, a genetic neurodevelopmental disorder with mutations in the X-chromosomal *MECP2* (methyl-CpG-binding protein 2) gene, affecting mostly girls, is inflicted with multiple mitochondrial dysfunctions, including brain-region specific (neocortex) reduction in 14-3-3 protein theta [86]. In this respect, the ADNP syndrome, at least in one case study, was originally misdiagnosed for the Rett syndrome [87]. Important for brain function [88], 14-3-3 serves as an ADNP shuttling protein between cellular compartments linked with sex differences in calcium influx [23], together offering common underlying mechanisms in neurodevelopment [89, 90].

Lastly, many protocadherins (pcdhs), members of a cellular adhesion protein family involved in synaptogenesis, neuronal survival, and neurodevelopmental regulation [91] were differentially expressed due to the Tyr mutation (Table S1). Most notably *Pcdhga11* is up-regulated in response to multiple ADNP mutations and retained differential expression in Tyr-mice undergoing NAP treatment. The complex role of Gamma-protocadherins (Pcdhgs) extends to vascular endothelial cells [92] and neocortical fine-structure [93]. The synaptogenic role of protocadherins is directly related to the critical effect of ADNP on dendritic spine formation [21], which exhibits sexual dimorphism that is accentuated in the Tyr mice (i.e., with the most significant mutation effect being seen in the male hippocampus corrected by NAP treatment, versus the most significant mutation effect seen in the female motor cortex, which is implicated in more extensive gait aberrations than in males, and is corrected by NAP treatment [22]). Pcdhgs are regulators of cortical interneuron programmed death (Bax-dependent [94]), while down-regulation of *Pcdhga11* was detected in the hippocampus of a learned helplessness rat model [95]. Pcdhg expression is also essential for maturation of newborn SVZ granule cells [96].

Genes encoding Pcdhgs that were shown here to be differentially expressed are positive regulators of WNT (i.e., *Pcdhga8*, *Pcdhga9*, *Pcdhgb1* and *Pcdhgc5*), except for the neutral *Pcdhga11* [97]. As indicated in the Introduction, Adnp prevents β-catenin degradation by binding its armadillo domain through the NAP motif, thereby disassociating β-catenin from the AXIN + APC degradation complex, enhancing WNT signaling and promoting neural induction/neurogenesis [7]. Hence, the interaction between ADNP/NAP and Pcdhg is multifunctional, acting through expression regulation, as well as protein interaction/WNT signaling pathway.

In terms of WNT signaling, neurotrophic factor (NT)-α1 (also known as carboxypeptidase E, CPE) was suggested as a key Wnt-β-catenin dependent anti-proliferation factor and ERK-Sox9 activated inducer of embryonic neural stem cell differentiation to astrocytes in neurodevelopment [98] on the one hand, and as important factor for cognition and Alzheimer’s disease, on the other hand [99], indicating converging pathways with ADNP.

Continuing with sexual differences and brain circuitry regulation, rare genetic variants in the *PCDH* gene family, including *PCDHGA11*, were recently discovered in a cohort of transgender women [100]. With ADNP regulating PCDHGA11, as well as sex steroid hormones [9], female-specific mitochondrial genes, and with mitochondria involved in early sex orientation [101], as well as ADNP modulating blastocyte formation, it is inferred that ADNP-mediated regulation is essential for the initiation of embryogenesis [102], a process further associated with sexual brain determination and affecting sex-dependent neurogenesis, as described here.

In conclusion, we have discovered, for the first time male, ADNP-specific regulation of hippocampal gene expression through the UPR, a process important for neurogenesis in a sex-dependent manner. Our results are in complete agreement with those presented by Budny et al. [64] who found sex-specific regulation of heat shock UPR genes as central regulators of sex differences in mouse hippocampal development. We, moreover, showed an up-stream control of ADNP as a master regulator of brain growth. We maintain that ADNP, and possibly other autism-related genes, not only regulate learning, memory, and social behavior, but also sex-related development and behaviors. Our results thus contribute to a comprehensive understanding of sex-dependent brain molecular structure and function.

## Supplementary information

Supplemental Material

Table S1

Table S2

Table S3

## Data Availability

The original sequencing data used in this study has been deposited in the NCBI Gene Expression Omnibus (GEO) database under accession code GSE272331. All other relevant data, such as gene-sets and genome references are publicly available for download.

## Code availability

The source code for the reproducible workflow is available upon request.
