# Supplementary material for: ADNP is essential for sex-dependent hippocampal neurogenesis, through male unfolded protein response and female mitochondrial gene regulation
Source: Mol Psychiatry. 2024 Dec 23;30(6):2696–706. doi: 10.1038/s41380-024-02879-w (PMC12092271; doi:10.1038/s41380-024-02879-w)
Supplement: Supplementary file 1 — Supplemental Material [file 41380_2024_2879_MOESM1_ESM.docx]

**Supplemental Materials**

**ADNP is essential for sex-dependent hippocampal neurogenesis, through male unfolded protein response and female mitochondrial gene regulation**

Guy Shapira^1,^*, Gidon Karmon^2,^*, Gal Hacohen-Kleiman^2^, Maram Ganaiem^2^, Shula Shazman^3^, Paschalis Theotokis^4^, Nikolaos Grigoriadis^4^, Noam Shomron^1^ and Illana Gozes^2,^**

^1^ Department of Cell and Developmental Biology, Faculty of Medical and Health Sciences, Sagol School of Neuroscience, Edmond J Safra Center for Bioinformatics, Tel Aviv University, Tel Aviv 6997801, Israel

^2^ Elton Laboratory for Molecular Neuroendocrinology, Department of Human Molecular Genetics and Biochemistry, Faculty of Medical and Health Sciences, Adams Super Center for Brain Studies and Sagol School of Neuroscience, Tel Aviv University, Tel Aviv 6997801, Israel

^3^ Department of Mathematics and Computer Science, The Open University of Israel, Ra'anana 4353701, Israel

^4^ Department of Neurology, Laboratory of Experimental Neurology, AHEPA University Hospital, Aristotle University of Thessaloniki, Thessaloniki, Greece

*Contributed Equally

**Address correspondence to: Professor Emerita Illana Gozes, The former Lily and Avraham Gildor Chair for the Investigation of Growth Factors, Head, The Elton Laboratory for Molecular Neuroendocrinology, Department of Human Molecular Genetics and Biochemistry, Faculty of Medical and Health Sciences, The Adams Super Center for Brain Studies and Sagol School of Neuroscience, Tel Aviv University, Tel Aviv 69978, Israel; [igozes@tauex.tau.ac.il](mailto:igozes@tauex.tau.ac.il)

**Running title:** ADNP determines sex-dependent neurogenesis

**Supplemental methods**

**BrdU immunohistochemistry**

Fixed tissue sections were incubated overnight with primary antibodies against rat BrdU (ab6326, Abcam, Cambridge, UK; 1:800), followed by a two-hour incubation with secondary antibodies (goat anti-rat Alexa Fluor 555-conjugated a21434; Invitrogen, Waltham, MA; 1:500). All cell counting (cells/mm^2^) was conducted by two different investigators, blind to the experiment, in six areas per section (near the lateral ventricles), spaced at least 50 μm apart. Only Dapi+ and BrdU+ nuclei were considered. Images were captured using an Axioplan- 2 optical/widefield fluorescent microscope (Zeiss, Oberkochen, Germany) while confocal images were captured with a Nikon Eclipse Ti microscope and assembled using ImageJ (Fiji) software. Statistical analysis was performed using Sigmaplot (Grafiti, CA) as detailed in the legend of Fig. 1.

**Bioinformatics analysis**

The batch effect was corrected using surrogate variables derived from the sva package^1^, used as model covariates in DESeq2^2^, or applied to the count data using limma^3^. Gene-level analysis was performed using DESeq2, including normalization of expression levels, differential expression tests and false discovery rates correction. Differential transcript expression levels were further tested with the Swish package^4^, controlling for uncertainty by generating 20 inferential replicates via Gibbs sampling. Heatmaps were created using the complexHeatmaps package^5^, with colors restricted to a reduced log2-fold change range, due to dynamic range considerations. To make the log2-fold change values more representative of the magnitude of differential gene expression, we used a transformed value, produced by the empirical bayes method ashr^6^. Enrichment testing was performed using the set-overlap and pre-ranked functions of the GSEApy package ^7^, with gene-sets from Enrichr^8^ and rummagene^9^. All gene-sets mentioned in this paper had significant enrichment results in one or more comparisons. Genotyping was performed according to the GATK best practices workflow for RNAseq short variant discovery^10^. In short, processed reads were aligned to the GRCm38 reference genome using STAR^11^ (two pass alignment), followed by post-processing by picard and variant calling with GATK. The reproducible workflow is linked in the code availability section.

**Supplementary excel tables**

- Table S1: Differential gene expression results (FDR<0.1), excluding pseudogenes and genes with low expression (mean normalized expression < 10).
- Table S2: Differential transcript expression results

Table S3: Results of gene set enrichment analyses (GSEA) and over-representation enrichment using Rummagene

**References:**

1 Leek, J. T., Johnson, W. E., Parker, H. S., Jaffe, A. E. & Storey, J. D. The sva package for removing batch effects and other unwanted variation in high-throughput experiments. *Bioinformatics* **28**, 882-883 (2012). https://doi.org:10.1093/bioinformatics/bts034

2 Love, M. I., Huber, W. & Anders, S. Moderated estimation of fold change and dispersion for RNA-seq data with DESeq2. *Genome Biol* **15**, 550 (2014). https://doi.org:10.1186/s13059-014-0550-8

3 Ritchie, M. E. *et al.* limma powers differential expression analyses for RNA-sequencing and microarray studies. *Nucleic Acids Res* **43**, e47 (2015). https://doi.org:10.1093/nar/gkv007

4 Zhu, A., Srivastava, A., Ibrahim, J. G., Patro, R. & Love, M. I. Nonparametric expression analysis using inferential replicate counts. *Nucleic Acids Res* **47**, e105 (2019). https://doi.org:10.1093/nar/gkz622

5 Gu, Z. & Hubschmann, D. Make Interactive Complex Heatmaps in R. *Bioinformatics* **38**, 1460-1462 (2022). https://doi.org:10.1093/bioinformatics/btab806

6 Stephens, M. False discovery rates: a new deal. *Biostatistics* **18**, 275-294 (2017). https://doi.org:10.1093/biostatistics/kxw041

7 Fang, Z., Liu, X. & Peltz, G. GSEApy: a comprehensive package for performing gene set enrichment analysis in Python. *Bioinformatics* **39** (2023). https://doi.org:10.1093/bioinformatics/btac757

8 Kuleshov, M. V. *et al.* Enrichr: a comprehensive gene set enrichment analysis web server 2016 update. *Nucleic Acids Res* **44**, W90-97 (2016). https://doi.org:10.1093/nar/gkw377

9 I-TASSER server for protein structure and function prediction. Avialable online: https://zhanggroup.org/I-TASSER (accessed on 20/12/2023).

10 Brouard, J. S. & Bissonnette, N. Variant Calling from RNA-seq Data Using the GATK Joint Genotyping Workflow. *Methods Mol Biol* **2493**, 205-233 (2022). https://doi.org:10.1007/978-1-0716-2293-3_13

11 Dobin, A. *et al.* STAR: ultrafast universal RNA-seq aligner. *Bioinformatics* **29**, 15-21 (2013). https://doi.org:10.1093/bioinformatics/bts635
